# Glioblastoma: Emerging Treatments and Novel Trial Designs

**DOI:** 10.3390/cancers13153750

**Published:** 2021-07-26

**Authors:** Vincenzo Di Nunno, Enrico Franceschi, Alicia Tosoni, Lidia Gatto, Raffaele Lodi, Stefania Bartolini, Alba Ariela Brandes

**Affiliations:** 1 Department of Oncology, AUSL Bologna, Via Altura 3, 40139 Bologna, Italy; e.franceschi@ausl.bologna.it (E.F.); a.tosoni@ausl.bologna.it (A.T.); lidia.gatto@ausl.bologna.it (L.G.); stefania.bartolini@ausl.bologna.it (S.B.); alba.brandes@ausl.bologna.it (A.A.B.); 2 Istituto delle Scienze Neurologiche di Bologna, Istituto di Ricovero e Cura a Carattere Scientifico (IRCCS), 40139 Bologna, Italy; raffaele.lodi@isnb.it

**Keywords:** glioblastoma, newly diagnosed glioblastoma, recurrent glioblastoma, GBM, new trial design

## Abstract

**Simple Summary:**

Nowadays, very few systemic agents have shown clinical activity in patients with glioblastoma, making the research of novel therapeutic approaches a critical issue. Fortunately, the availability of novel compounds is increasing thanks to better biological knowledge of the disease. In this review we want to investigate more promising ongoing clinical trials in both primary and recurrent GBM. Furthermore, a great interest of the present work is focused on novel trial design strategies.

**Abstract:**

Management of glioblastoma is a clinical challenge since very few systemic treatments have shown clinical efficacy in recurrent disease. Thanks to an increased knowledge of the biological and molecular mechanisms related to disease progression and growth, promising novel treatment strategies are emerging. The expanding availability of innovative compounds requires the design of a new generation of clinical trials, testing experimental compounds in a short time and tailoring the sample cohort based on molecular and clinical behaviors. In this review, we focused our attention on the assessment of promising novel treatment approaches, discussing novel trial design and possible future fields of development in this setting.

## 1. Introduction

Glioblastoma (GBM) is the most common primary brain tumor, with an estimated incidence of 3.22/100,000 persons in the United States and a five-year overall survival of only 6.8% [1,2]. Nowadays, GBM can be diagnosed as a diffuse astrocytic glioma without IDH and H3R gene mutations, with microvascular proliferation, necrosis, and/or peculiar molecular features such as TERT mutation, EGFR amplification, and/or gain of chromosome 7 combined with the loss of chromosome 10 [3,4,5,6]. According to the EANO guidelines for the diagnosis and treatment of diffuse gliomas of adulthood, isocitrate dehydrogenase (IDH)-mutated glioblastoma should be better defined as a grade 4 IDH-mutant astrocytoma [6].

Current management of patients with GBM employs maximal safe resection surgery followed by radiation and chemotherapy [2,7,8,9,10].

Recurrent GBM can be managed by different approaches [11,12,13], including loco-regional treatment and systemic treatments [2,14,15,16,17,18,19].

The prognosis of patients with GBM remains poor, with an estimated overall survival (OS) of 12–18 months from primary diagnosis and a life expectancy of 5–10 months after the diagnosis of recurrent GBM [20,21,22]. 

Since treatments provided are not curative, guidelines strongly recommend the patient’s inclusion in clinical trials [2,23].

In the last decade, several novel discoveries about the molecular, genomic, and biological background of the disease have been determined. Nonetheless, none of these improvements translated into a significant progress in terms of therapeutic options. Indeed, several drugs and approaches showing promising results in early studies failed to confirm a clinical improvement on large randomized trials. Furthermore, the enrollment on clinical trials is limited, with only 10% of GBM patients being enrolled in a clinical study [24,25]. 

The purpose of the present paper is to investigate possible reasons related to the lack of therapeutic improvements on GBM, focusing on possible improvements in terms of trial planning and design. We also reviewed more promising experimental systemic treatments for patients in early phase of development, as well as in patients with newly diagnosed and recurrent GBM.

## 2. Therapeutic Targets on GBM

Several biological obstacles make the development novel effective drugs difficult [26,27]. These are represented by (1) the blood–brain and blood– tumor - brain barrier which makes the passage of therapeutic compounds difficult, (2) the extreme heterogeneity of the disease, and finally (3) the capacity to develop molecular mechanisms able to promote treatment resistance to antitumoral treatment. All these elements reduce the development of novel target agents. Nonetheless, the increasing knowledge of the molecular mechanisms related to disease development and progression has allowed the identification of several attractive targets for the systemic management of GBM (Table 1) [26,27]. The majority of these targets are represented by tyrosine kinase (TK) receptors. 

The amplification of the epidermal growth factor receptor can be found in about 50% of GBMs [28], and several agents targeting this pathway have been investigated in GBMs (a discussion of treatments proposed for EGFR inhibition is included in Section 4. Recurrent GBM). Other than EGFR, some other TK receptors have gained particular interest.

Altered tumor vascularization is one of the hallmarks of the disease and there are at least two TK receptors whose inhibition could be associated with angiogenesis regression and tumor responses. These are the vascular endothelial growth factor receptor (VEGFR) and the platelet-delivered growth factor receptor (PDGFR) [16,29]. Several small TK inhibitors (TKIs) targeting one or both of these two receptors have been tested without significant benefit [16]. Imatinib, pazopanib, cediranib, sunitinib, sorafenib, nintedanib, tivozanib, dovitinib, crenolanib, and cabozantinib are all oral TKIs that failed to show a significant clinical benefit on patients with GBM [16]. The mesenchymal–epithelial transition (MET) receptor is another pathway that could be activated in GBM cells [30]. Although the multi-target and MET inhibitor cabozantinib showed only a modest effect on GBM [31] (weighted by a high adverse events rate), the oral MET inhibitor capmatinib is under investigation for patients with GBM, in combination with bevacizumab (NCT02386826).

The epidermal growth factor receptor 2 (HER2) amplification is a driver molecule that is well-targeted by several target compounds in breast cancer. This receptor can be amplified also in GBM cells [32]. However, to date, no agents targeting HER2 have shown clinical efficacy on patients with GBM. Indeed, the oral inhibitors lapatinib and neratinib failed to show a significant impact on patients with GBM and in patients with brain metastases from solid tumors [33,34]. The novel oral TKI tucatinib has been shown to pass through the blood–brain barrier, reaching therapeutic concentrations in the brain [35]. Although this could be an effective treatment on patients with HER2-altered GBM, no trials are investigating this agent. 

The management of several solid tumors has been revolutionized by the advent of immune-checkpoint inhibitors. Briefly, these agents can restore an inhibited immune response against tumors and are effective also on brain metastases from solid malignancies [36]. Their role will be further discussed in the next paragraph. Nonetheless, some other immunological approaches are assuming particular interest in the hematological and solid tumor treatment field [37]. Chimeric antigen receptor T cells (CAR-Ts) and chimeric antigen receptor macrophage (CAR-Ms) are surely two of the most enthusiastic approaches, involving the genomic recombination of T cells or macrophages which are oriented against tumor cells. Although there is little data regarding the safety and efficacy of this approach on GBM, several early phase studies are assessing this strategy on patients with GBM (NCT04077866, NCT04741984). Nonetheless, it is still unclear which could be the optimal cell manufacture and administration process. Of interest, some data are suggesting that CAR-Ms could be key strategies for GBM management, mainly thanks to the better penetration of the macrophage into the tumor-associated microenvironment (NCT04741984) [38]. 

Indoleamine 2,3-dioxygenase (IDO) 1 and 2 are catabolic enzymes involved in the degradation of tryptophan. IDO is supposed to promote a negative regulation of immune response, and it has the potential to inhibit both innate and adaptive responses against the tumor [39]. Although these agents have been already tested on GBM, the combinations of IDO inhibitors such as indoximod, epacadostat, BMS-986205, [40,41] and immune checkpoint, chemotherapy, and/or radiation treatment is under assessment in several different trials (NCT04047706).

Oncolytic viruses are reprogrammed viruses able to specifically target tumor cells, replicating and killing them [42,43,44,45]. Previous studies suggested a potential effective role of these agents against GBM in preclinical models and within early clinical studies [42,43,44,45]. Thus, several trials are testing these agents on GBM patients (NCT03294486, NCT03714334, NCT02062827). 

## 3. Newly Diagnosed GBM 

Since 2005, the post-surgical standard treatment of GBM is surgical resection followed by temozolomide (TMZ), concomitant with and adjuvant to radiotherapy (60 Gy over six weeks), leading to a median survival time of 14.6 months [9]. The benefit from TMZ is greater in patients who present MGMT promoter methylation, which epigenetically silences the gene [7]. 

Different improvements of the current protocol have been tested in recent years. 

Two trials demonstrating an improvement in overall survival with standard treatment have not been fully incorporated in the actual therapeutic scenario for different reasons. Tumor-treating fields (TTFields) is an antimitotic treatment modality, which acts by delivering a low-intensity (200 kHZ) electric field within the brain, alternating electric fields to the tumor. Through this action, TTFields interferes with GBM cell division and organelle assembly. The efficacy of incorporating TTFields in the standard first line treatment has been explored in the EF-14 trial [46]. In this randomized trial, 695 GBM patients, after completed concomitant radio chemotherapy, were randomized to TTFields plus maintenance TMZ or TMZ alone. The addition of TTFields lead to a significant increase in PFS (6.7 vs. 4.0 months, HR, 0.63; 95% CI, 0.52–0.76; *p* < 0.001) and OS (20.0 vs. 16.0 months HR, 0.63; 95% CI, 0.53–0.76; *p* < 0.001) over standard treatment, without significant difference in adverse events. Despite the FDA approving TTFields for newly diagnosed GBM in 2015, the use in clinical practice remains limited (3–12% of patients with newly diagnosed GBM) due to patients declining to wear the device, combined with difficulty in understanding the mechanism of action, doubts about the favorable outcome of existing studies, and the high costs of the treatment (to date, this treatment strategy is mainly adopted by USA, Israel, and Switzerland).

The CeTeG/NOA-09 German trial has randomized 141 MGMT-methylated GBM patients to standard TMZ concomitant with and adjuvant to radiotherapy, or to six cycles of a lomustine and TMZ combination in addition to radiotherapy [47]. Median OS was 31.4 months in the TMZ group, compared to 48.1 months in the lomustine–TMZ group (hazard ratio (HR) 0.60; 95% CI, 0.35–1.03; *p* = 0.0492). There was no difference in terms of progression-free survival (PFS), while adverse events of grade 3 or higher were observed in 51% and 59% of patients in the TMZ group and lomustine–TMZ group, respectively. However, the study presents some significant limitations. First, the small cohort of patients limits the validity of the results and presents the possibility of biases. Furthermore, the low number of randomized patients is in contrast with the high number of MGMT-methylated screened patients, with an accrual rate of only 60%. Another interesting issue was the improvement in OS which was not associated to a PFS benefit. This was not observed in previous newly diagnosed GBM phase III trials [9,46], and was not explained by differences in subsequent treatments at recurrence/progression.

Moreover, no survival benefit has been demonstrated with TMZ dose-dense regimens [48] or with extension of maintenance treatment up to 12 cycles [49]. To further explore this setting, the ANOCEF group proposes a randomized trial (NCT03663725) comparing standard treatment versus an intensified arm consisting of one TMZ cycle started between day 2 and 15 after surgery, followed by TMZ concomitant to radiotherapy, followed by maintenance TMZ until progression, intolerance, the patient’s or the physician’s decision.

Given the potential role of hypoxia in the biology of GBM, the addition of antiangiogenic therapy with bevacizumab has been investigated in two large phase III randomized trials in the first line setting [50,51]. Despite prolonging PFS in both trials, the addition of bevacizumab failed to demonstrate an overall survival improvement. Moreover, bevacizumab was associated with an increase in adverse events.

The introduction of immune checkpoint inhibitors (ICIs) has recently revolutionized the therapeutic scenario in a number of different cancer types. ICIs act as inhibitors of immune-checkpoints, restoring an inhibited immune-response against the tumor. Two phase III clinical trials investigated nivolumab (a programmed death receptor-1 (PD-1) inhibitor) in combination with radiation therapy in patients with unmethylated MGMT GBM (CheckMate-498; NCT02617589), and in association with radiation therapy plus concomitant and adjuvant temozolomide in patients with methylated MGMT glioblastoma CheckMate-548; NCT02667587). Unfortunately, none of these trials showed significant improvement in terms of OS and PFS for patients receiving nivolumab.

Another immunotherapy first line phase II trial, PERGOLA (NCT03899857) is evaluating the addition of pembrolizumab to standard treatment in newly diagnosed GBM patients. 

The ICIs combination with ipilimumab and nivolumab has been initially studied in exploratory phase I cohorts. In these patients there was a significant rate of high-grade adverse events, with a discontinuation rate due to toxicity accounting for 20.30 [52]; thus, this combination strategy has not being further assessed in the subsequent phase III trials. The ICI ipilimumab and nivolumab combination is now being retested in a phase II/III study in newly diagnosed MGMT unmethylated GBM patients, comparing the usual treatment with radiation therapy and TMZ to radiation therapy in combination with ipilimumab and nivolumab (NCT04396860). 

The role of active immunotherapy via vaccine injection is being explored in the ongoing study of dendritic cell (DC) immunotherapy against cancer stem cells. In this study, newly diagnosed GBM patients are vaccinated during standard treatment with ex vivo generated DCs transfected with mRNA from autologous tumor stem cells, survivin, and hTERT.

Enzastaurin (enz) inhibits protein kinase C-beta, angiogenesis, and has a direct cytotoxic activity against glioma cells [53]. Previous phase II studies carried out in recurrent high-grade glioma and in newly diagnosed MGMT unmethylated GBM patients did not show any significant single-agent activity [54,55]. However, the recent discovery of a novel biomarker, de novo genomic marker 1 (DGM1), a germline polymorphism on chromosome 8, highly correlated with response to enz in both lymphoma and GBM [56], has prompted the clinical development of this drug. In particular, GBM patients with DGM1+ assessment receiving enz had a median OS of 18 months versus 12.8 months in DGM1− patients (HR 0.68; 95% CI, 0.25–1.81; *p* = 0.12). Given these data, a randomized double-blind, placebo-controlled phase III study of enz added to temozolomide during and following radiotherapy in newly diagnosed GBM with or without DGM1 has been recently launched in the US (NCT03776071).

The phase III trial EORTC 1709 evaluating the addition of marizomib, a novel brain-penetrant pan-proteasome inhibitor, to standard TMZ/RT→TMZ in newly diagnosed GBM has been prematurely closed by IDMC, after evidence of more frequent grade 3/4 treatment-emergent adverse events compared to the standard therapy group (42.6% vs. 20.5%), including ataxia, hallucinations, and headache. The study did not show a significant impact on OS or PFS over standard treatment in [57].

Adaptive platform trials allow the testing of several experimental drugs at the same time, developing a more efficient and cost-effective mechanism for accelerating treatment approval for patients. In the neuro-oncology field, the GBM AGILE study (NCT03970447) is evaluating several experimental compounds on patients with newly diagnosed and recurrent GBM, tailoring each experimental arm according to the molecular assessment of the disease. AGILE opened for patient enrollment in 2019, and site activation is ongoing in the US, whereas expansion to Canada, Europe, and China are under progress. The trial is evaluating a new treatment arm using regorafenib, paxalisib, and VAL-083 in maintenance period in newly diagnosed GBM after concomitant treatment [58].

Despite available treatments, GBM inevitably recurs, demonstrating a poor overall prognosis with a two-year survival rate of less than 20%. Nevertheless, it should be highlighted that a small proportion of patients achieve a long survival of over three years, but the molecular prognostic and predictive background dividing long-term (LTS) from short-term survivors (STS) is still poorly understood. Nonetheless, some studies investigated the clinical and molecular behaviors of LTS. Overall, LTS were younger at diagnosis, female, and presented MGMT methylation. The sphingomyelin metabolism was also increased in these patients [59,60,61]. With the aim to understand biological background of LTS, EORTC is conducting the EORTC 1419 Eternity trial (NCT03770468). This prospective and retrospective multicentric clinical epidemiological study will evaluate the molecular genetics, and host-derived and clinical determinants of GBM patients with an overall survival of more than five years.

## 4. Recurrent GBM

Effective treatment options are limited, and new therapeutic strategies are desperately needed. As of yet, nitrosoureas are still considered the standard of care for recurrent GBM. Several tyrosine kinase inhibitors (TKIs) and monoclonal antibodies (mAbs) targets have been investigated in the last few years with limited results [14,15,17,18,62,63,64,65], while many others are in clinical development in recent clinical trials.

About 50% of all GBM patients present an amplification of the epidermal growth factor receptor (*EGFR*) gene which represents a driver mutation in GBM. Most frequent EGRF mutations are represented by EGFRA289D, EGFRA289T, and EGFRA289V [28]. Nonetheless, agents targeting this receptor failed to show a significant survival impact on patients with GBM [66,67,68].

Recently, depatuxizumab mafodotin (depatux−m, ABT414), an antibody-drug conjugate that consists of an antibody directed against EGFR and EGFRvIII, conjugated to a toxin (monomethyl auristatin F), was evaluated in the INTELLANCE-2/EORTC_1410 randomized phase II study [69]. Patients receiving depatux-m and TMZ had a trend towards improved survival (primary analysis: HR 0.71; 95% CI, 0.50–1.02; *p* = 0.06; second follow up analysis: HR 0.66; 95% CI, 0.48–0.93; *p* = 0.024), corresponding to a median OS difference of 9.6 months (deatux-m + TMZ) versus 8.2 months (TMZ). The presence of EGFR single-nucleotide variations (SNVs) was shown to predict an improved outcome in the depatux−m + TMZ arm. These SNVs result in a receptor that is hypersensitive to low-affinity EGFR ligands, which can explain the increased activity of depatux−m and TMZ [70].

Antiangiogenic approaches have been investigated since 2007, with bevacizumab being the most studied agent [14,19,71,72,73,74,75,76,77,78]. Despite promising results in terms of progression-free survival across multiple studies, these results did not translate into an overall survival benefit in the randomized phase III EORTC 26101 trial that compared bevacizumab and lomustine with lomustine alone (9.1 vs. 8.6 months, hazard ratio for death, 0.95; 95% CI, 0.74–1.21; *p* = 0.65)

More recently, regorafenib, an oral multi-kinase inhibitor targeting VEGFR-1, -2, -3, TIE 2, PDGFR, FGFR, KIT, RAF-1, RET, and BRAF has been investigated in the randomized phase II trial REGOMA, which has been approved for the management of recurrent glioblastoma by the EMA (European Medicines Agency) [17]; this trial showed a median OS of 7.4 months in the regorafenib arm vs. 5.6 in the lomustine arm. Thus, this agent has been included in other ongoing trials (i.e., the AGILE study) or in combination with other agents (i.e., Nivolumab, NCT04704154). Alteration of the cyclin-dependent kinase 4–6 (CDK4–6) pathway is a common event in GBM. A phase II trial evaluated the role of palbociclib in recurrent GBM patients with RB1 proficiency. Despite adequate penetration in tumor tissue, palbociclib showed limited activity with a median PFS of 5 weeks and a median survival of 15.4 weeks [79]. Similarly, in another phase II trial in patients with recurrent GBM and with evidence of CDKN2A/B loss and intact RB, abemaciclib showed a six-month PFS of 9.37% (95% CI, 2.4–22.7%), a median PFS was 55 days (95% CI, 49–56 days), and a median OS of 384 days (95% CI, 228–488). 

Larotrectinib is a selective TRK inhibitor that showed an impressive response rate and also durable disease control in GBM patients. The study [80], presented at the 2019 ASCO meeting, evaluated 18 cases with primary brain tumors, including six (32%) patients with GBM. A disease control rate was achieved in 100% of patients (in 14 evaluable patients), with a disease control rate ≥16 and 24 weeks in 79% and 71% of patients, respectively; the median PFS was 11 months (95% CI, 2.8–Not Reached). At the recent 2021 ASCO meeting, data regarding larotrectinib suggested that better results were obtained in pediatric patients with brain tumors, while no partial responses were seen in adult glioma patients.

Agents targeting BRAF inhibit the downstream altered MAPK pathway, which is often altered in solid tumors and is also an important driver of cell proliferation in glioma patients. V600E is the most frequent mutation in the BRAF gene described in gliomas, occurring in about 5% of adults [81]. Vemurafenib and dabrafenib, selective oral tyrosine kinase inhibitors of the oncogenic BRAF V600 kinase, have been tested in BRAF mutant melanoma patients. The role of vemurafenib in BRAF V600-mutant gliomas has been investigated in the VE-BASKET trial [82], which evaluated 24 patients (six GBM, five anaplastic astrocytoma, one high grade glioma not otherwise specified, and twelve with other histologies). For high-grade glioma patients, the response rate was 9%, the median PFS was 5.3 months, and the median survival was 11.9 months. Combined inhibition of BRAF and MEK in gliomas was also investigated in the ROAR basket trial [83]; in the group of high-grade gliomas, response rate was 27%, and the disease control rate was 57%.

Immunotherapies have also been investigated in recurrent GBM. The Check-Mate-143 trial evaluating nivolumab (a PD-1 inhibitor) versus bevacizumab in recurrent GBM was negative in the general population [18]. Nonetheless, the response duration was longer in the nivolumab (11.1 months) arm as compared to the bevacizumab arm (5.3 months). The corticosteroid use did not impact survival in the bevacizumab arm, while reduced doses were associated with an improved clinical outcome in the nivolumab treatment arm (HR, 0.59; 95% CI, 0.36–0.95). Moreover, a trend toward a longer survival was observed in MGMT-methylated patients without any baseline corticosteroids receiving nivolumab over bevacizumab (17.0 vs. 10.1 months; HR, 0.58; 95% CI, 0.30–1.11). 

Pembrolizumab was also evaluated as a “neoadjuvant” treatment for recurrent GBM in an Ivy Foundation Early Phase Clinical Trials Consortium randomized study. Cloughesy and Colleagues evaluated the survival and immune response obtained when using pembrolizumab before and/or after surgery in 35 recurrent GBM patients [84]. Patients in the “neoadjuvant” arm with continued adjuvant therapy following surgery reported a significant increase in survival compared to patients treated with pembrolizumab only after surgery, with a median survival of 13.7 months in the “neoadjuvant/adjuvant” arm vs. 7.5 months in the “adjuvant”-only arm (HR: 0.39; 95% CI, 0.17–0.94; *p* = 0.04). Interestingly, treatment with pembrolizumab before surgery was associated with upregulation of T cell- and interferon-γ-related gene expression, but downregulation of cell cycle-related gene expression within the tumor. 

Another immunotherapy approach consists of vaccination against EGFRvIII, a GBM-specific EGFR driver mutation [85]. Rindopepimut in combination with bevacizumab, or a control injection of keyhole limpet hemocyanin in combination with bevacizumab, were investigated in a randomized phase II trial in recurrent EGFRvIII-positive GBM patients. The primary endpoint was PFS at six months, which was 28% for rindopepimut and 16% for the control (*p* = 0.12); the analysis of survival, a secondary endpoint, showed a statistically significant advantage in the rindopepimut–bevacizumab arm (HR 0.53; 95% CI, 0.32–0.88; *p* = 0.01). Additionally, in a randomized phase III study investigating rindopepimut in patients with newly diagnosed GBM, this agent did not improve OS compared to the standard of care [86].

Another immunotherapy approach consists of active immunization (i.e., dendritic cells or peptide vaccines). Dendritic cells (DCs) are antigen-presenting cells able to induce adaptive immunity. Due to promising results from a phase III trial in a newly diagnosed setting [87] with an autologous tumor lysate-pulsed dendritic cell vaccine (DCVax^®^-L), similar approaches are now under investigation in phase III trials in the recurrent setting GBM (NCT04277221).

## 5. Problematic Issues on Interventional Trials: The Glioblastoma Paradox

The lack of therapeutic improvements in the last years appears even more disappointing considering the increasing scientific understanding of the disease and the large availability of novel potential active compounds to test. 

This paradox makes GBM a unique disease in which the availability of key molecular and biological insight does not translate into the development of new drugs.

The presence of the blood–brain barrier, the heterogeneous and complex biology of the disease, and the lack of sufficient investment are possible explanations of this failure. 

Nonetheless, some concerns emerge about the modality by which these novel compounds are tested therefore the clinical trial landscape (Table 2).

In 2018, Vanderbeek A.M. et al. published the results of a survey of clinical trials reported on clinicaltrials.gov, including GBM patients in the United States from 2005 to 2016 [25]. Interestingly, they reported over 400 clinical trials of which the majority were represented by phase I/II and phase II studies (60%) [25]. 

Of note, the authors found a very high rate of uncompleted and terminated trials with one to ten studies concluded due to lack of accrual, funding, or futility (no clinical advantage emerging at early assessment) [25]. Moreover, there was a median time to study completion of three to four years in phase II studies. These data appear even more surprising considering that only 5 of 249 phase I/II and phase II trials were randomized. Phase III trials were a minority, representing only 7% of all clinical trials assessed. Twelve of sixteen phase III trials were supported by a previous phase II study, and the overall population enrolled in these trials represented 26% of the total population assessed on clinical trials between 2005 and 2016 [25]. 

The authors concluded that only one to ten (8–11%) patients entered into clinical trials, which is a very frustrating result considering the rate of terminated trials due to lack of accrual [25].

Another well-known problematic issue related to interventional trials on GBM is the weakness of surrogate efficacy endpoints [50,51,95,99,100]. Indeed, progression-free survival (PFS) and overall response rate (ORR) is successfully adopted in clinical trials assessing novel compounds on solid malignancies as they provide a reliable prediction of other outcomes of interest, such as clinical improvement and overall survival (OS). The use of surrogate endpoints of OS could be important as they can reduce the time of the study. Nonetheless, the relationship between OS and surrogate such as PFS and ORR is extremely uncertain on GBM as survival benefit cannot reflect the improvement of PFS or ORR [50,51,95,99,100], especially in the case of antiangiogenic treatments. The post-progressive survival is a composite outcome, which has been assessed in a large series of over a thousand patients with GBM, and represents an interesting surrogate endpoint [101]. The research of reliable surrogates of OS acquires great importance in the assessment of novel agents in GBM.

The availability of novel potentially active drugs is increasing as biological and genomic assessment of the disease becomes even more clear. Furthermore, it has been demonstrated that GBM is not a unique disease as its molecular behaviors can drastically modify the clinical presentation, progression, and response to treatment. The larger the availability of novel compounds, the higher the need for interventional trials. This can be complicated considering the low incidence of the disease.

To date, only a few patients benefit from interventional clinical trials. The rate of terminated study due to lack of accrual is relatively high even if patients are strongly required, considering the increasing availability of novel agents. Additionally, the time to study completion is long, requiring years due to the absence of reliable surrogate endpoints of overall survival. Finally, the distribution of patients could be unbalanced, since the majority of them are enrolled in phase III trials with a relatively small number of patients enrolled in early phase I and II studies. This can lead to an unpowered early efficacy study, exposed to the risk of unclear information. The result is the early termination of potentially active compounds and a further unsuccessful test (on phase III) of unactive drugs. 

Excluding financial and biological problems related to the development of new effective compounds, these issues may represent a strong limitation to the clinical progress of GBM management.

### Improving Interventional Clinical Trials Design on GBM

The primary field of improvement is represented by organizational improvement and the need for investment in the research of active compounds, trial planning, and patients on trial tutelage [88,89,90]. 

Patients with GBM should be referred to reference centers and the development of inter-center networks providing early information about active trials should be encouraged. Similarly, the participation of patients in clinical trials could be encouraged through facilities allowing patient mobility, permanence in the experimental center during the trial course and follow up, and job and economic safeguarding of patients and caregivers. These elements could reduce the number of early terminated trials, as well as increase the number of patients who could benefit from a clinical trial (Table 2).

From the organizational point of view, there are several fields of improvement of clinical trials in GBM [88,89,90].

The introduction of a comparator arm in phase II study has provided a more accurate estimation of the efficacy of the novel compound under investigation [91], however, again, the transition from a positive randomized phase II [14,15,17,18,64,68,73,86,102,103,104,105] trial with a limited number of patients to a large phase III trial was negative [77]. Nonetheless, early randomized studies require more time for their completion and a higher number of patients as compared to single-arm phase II studies. To avoid these limitations, the incorporation of Bayesian statistics in trial design is a winning strategy [92]. Classical trials test a hypothesis among a distinct population, in a study with a pre-planned sample size dimension which conditions the power of the study. 

The hypothesis of the Bayesian model is not fixed, but its probability (for example to be true or false) is constantly modified during the study due to the increasing amount of data acquired. For example, the Bayesian adaptive randomized (AR) study can use the data accumulating in the course of the same trial to modify the treatment allocation according to the potentially more efficient interventional arms [92]. 

In 2012, Trippa L. et al. acquired data from different phase II trials assessing four different compounds. In their simulation, authors allocated these same patients into a Bayesian AR study, assessing the same interventional arms [92]. Results of this simulation were surprising, as the same findings of the previous phase II studies were confirmed without loss in statistical power and with a significantly lower number of patients required [92].

Nowadays, Bayesian AR is commonly adopted in clinical trial design and represents a significant improvement in terms of quality of the research due to the possibility of testing more treatments with a shared comparator arm, at the same time reducing the number of patients required.

Another commonly adopted strategy to overcome the need for a comparator arm, and thus the randomization, is the adoption of a historical cohort based on previous findings in clinical trials [91]. This strategy exposes the risk of several biases for different reasons. First, outcomes such as the survival of patients with GBM are not static values, as there is a trend showing increases in time even if there is no modification of treatment standards [91,106]. In addition, it has been well demonstrated that the inter-trial variability reflects a variable distribution of the outcome of interest, which significantly increases the risk of underestimating or overestimating the benchmarks [91]. This final result poses a very high risk of achieving false positive or negative observations in phase II trials, leading to a subsequent assessment of inactive compounds or the early termination of the study of an active drug [91].

Even if OS remains the best available clinical endpoint, the research of a novel surrogate endpoint is still a clinical need. 

The PFS improvement failed to show an improvement in OS across different clinical trials [50,51,95,99,100]. Nonetheless, PFS expressed as the rate of patients progressing at a specified interval of time is commonly adopted in GBM clinical trials [50,51,95,99,100]. Again, Bayesian AR trials can offer a possible solution to this problem [96]. Thanks to the flexibility of the Bayesian AR trial, the incoming data provided in the course of the clinical trial can allow early determination of whether concordance between OS and PFS exists, therefore allowing, in case of concordance, decision-making results based on the assessment of PFS alone [96]. 

There are several problems related to response assessment in patients with GBM [107,108,109,110]. Indeed, response assessment must involve other data in addition to dimensional and imaging criteria. The type of treatment provided and the molecular background of the disease are mandatory elements to estimate response to treatment. Integration of molecular and clinical data with imaging improves ORR estimation; nonetheless, functional imaging provided by magnetic resonance imaging (MRI) and positron emission tomography (PET) is increasing as to allow a more reliable distinction of progression/response to treatment [107,108,109,110,111,112,113]. Criteria of response assessment have been modified and reflects the type of treatment provided [107,108]. 

Novel technologies are currently employing the use of artificial intelligence algorithms which can, based on the data provided and learned, assess the disease [114,115], and improve the use of this endpoint.

Innovative trials on GBM are represented by AGILE, INSIGhT, and N2M2 trials [93,94,98].

The Adaptive Global Innovative Learning Environment (GBM AGILE) is a novel, multi-arm, platform trial which is composed of two different statistical designs [93]. The first phase is a Bayesian AR stage in which several compounds are tested with a common control. Through this phase, the aim is to isolate the active compounds and determine the population in which this is expected to be more effective, preventing and reducing the number of patients receiving ineffective treatments. Regarding this last point, results of the experimental arm investigating the CC-115 compound within the INSIGhT trial have been recently reported [116]. Thanks to the adaptive study design of the INSIGhT trial, a reduced number of patients received the experimental treatment which showed significant toxicity and lack of clinical efficacy [116]. Once that a promising active compound has been established, it proceeds to the second phase which involves classical fixed randomization to confirm the result of the Bayesian step [93]. Other advantages of this platform are represented by the inclusion of novel compounds at any time during the study. In addition, biomarkers can be assessed during each phase of the study allowing a fast discovery and validation of prognostic/predictive biological markers [93]. 

In addition, the INSIGhT trials employed a Bayesian AR in the first step [94]. Different from AGILE, in the INSIGhT trial, only patients with newly diagnosed unmethylated GBM without the isocitrate dehydrogenase (IDH) R132H gene mutation have been included. A key inclusion criterion is also represented by complete genomic data for biomarker groupings [94]. This trial is currently testing three different compounds simultaneously and comparing them to the standard represented by radiation and adjuvant temozolomide [94]. Preliminary results of the abemaciclib treatment arm have been recently reported showing no OS advantage for patients receiving the CDK inhibitor [117].

The paradigm of the precision medicine era is the administration of drugs tailored based on the biological background of tumor disease. AGILE and INSIGhT offer the possibility to test more drugs rapidly, isolating the population where the novel agent is more effective. 

The NCT Neuro Master Match (N2M2) offers a different solution, as the goal of this trial is to primarily identify the target population, then provide the drug which can result in a clinical improvement based on the biological background of the disease [98]. This is an umbrella trial for patients with unmethylated IDH wild-type GBM [98]. The design of the study is composed of two parts; the discovery phase provides a molecular and neuropathological assessment of the disease to detect predefined biomarkers for targeted treatments, while the treatment phase employs a stratification of the population based on the results obtained in the discovery phase. The Bayesian model is employed to provide continuous monitoring of toxicity in phase I, while the efficacy endpoint is represented by six-month progression-free survival [98].

Despite umbrella and molecular tailored designs being extremely attractive, it should be noted that GBM is a heterogeneous disease and that the isolation of potentially predictive biomarkers may not reflect a sensitivity to defined novel compounds.

**Figure 1:**
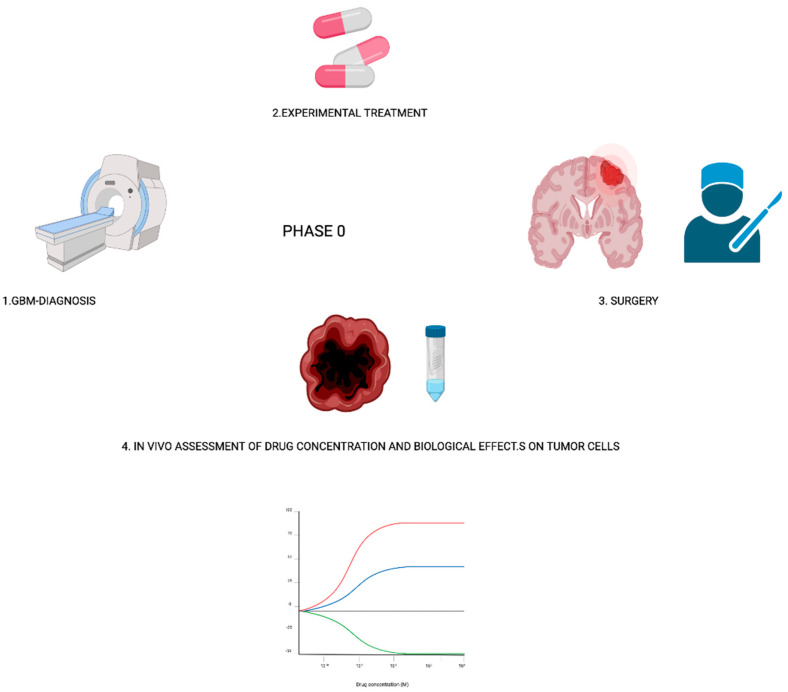
Phase 0 study overall design.

One proposed type of trial that specifically aims to target agents is represented by ‘’phase 0′’ studies [97] (Figure 1). 

Due to the protection offered by the blood–brain barrier (BBB) and the blood-tumor–brain barrier, several drugs failed to show a clinical effect on GBM. Phase 0 studies can rapidly assess pharmacological effects of the compounds on a patient’s tumors, also discovering if and how much the compounds pass the BBB and penetrate the tumor tissue. Briefly, the study design requires that patients assume the study drug one to two weeks before preplanned surgery. After surgery, there is an in vivo assessment of tumor tissue, cerebrospinal fluid, and/or blood. Vogelbaum M.A. et al. recently reviewed all phase 0 and phase 0-like studies carried out between 1993 and 2018, establishing that phase 0 study in neuro-oncology should include patients in which tumor resection is planned, and involve clinical doses of the investigational agent, a tissue sample from each part of the tumor (including enhancing and non-enhancing portions of the tumor), and the assessment of specific drug-related target effects [97].

## 6. Conclusions

Glioblastoma represents a clinical challenge for oncologists and researchers. The increasing knowledge of molecular mechanisms related to disease onset and progression has allowed the development of several novel compounds which should be assessed among clinical trials. The need to test more and more compounds at the same time led to the development of a next generation of trials adopting a Bayesian design. In addition, phase 0 trials can detect early and perform an in vivo assessment of drugs able to penetrate the tumor tissue, stopping further development of drugs unable to cross the blood–brain barrier. All these elements will surely contribute to the development of effective treatments against the disease, as well as to allow patients access to experimental compounds.

## Figures and Tables

**Table 1:** Clinical trials cited in the text. MGMT: methylation of the O(6)-methylguanine-DNA methyltransferase, TMZ: temozolomide.

Trial Name	Phase	Experimental Compounds	Setting
NCT02386826	I	Capmatinib and bevacizumab	Newly diagnosed and recurrent GBM
NCT04077866	I/II	B7-HR CAR-T	Glioblastoma cells expressing B7-H3
NCT04741984 DEMAND	I	Pp65CMV antigen monocytes	Newly diagnosed MGMT unmethylated GBM
NCT04047706	I	BMS-986205 + Nivolumab	Newly diagnosed GBM
NCT03294486 ONCOVIRAC	I/II	Combination of TG002 and 5-flucytosine	Recurrent GBM
NCT03714334	I	DNX-2440	Recurrent GBM
NCT02062827	I	M032-HSV1	Newly diagnosed GBM or recurrent GBM.
NCT03663725 StrateGlio	III	Intensified TMZ protocol	Newly diagnosed GBM
NCT03899857 PERGOLA	II	Pembrolizumab	Newly diagnosed GBM
NCT04396860	II/III	Ipilimumab + nivolumab	Newly diagnosed GBM-MGMT unmethylated
NCT03776071	III	Enzastaurin	Newly diagnosed GBM
NCT04704154	II	Regorafenib + nivolumab	Recurrent GBM
NCT04277221	III	Autologous Dendritic Cell/Tumor antigen	Recurrent GBM

**Table 2:** Challenges and innovations of trial design planning for patients with glioblastoma.

Challenges of Clinical Trials Design on GBM	Innovation Proposed
A small number of patients benefit from inclusion in clinical trials	The inclusion of patients should be encouraged through the development of inter-center networks and improvement of organizational phases. Investments in trial planning and facilities for patients enrolled in clinical trials can increase the number of patients in clinical trials [88,89,90].
Reduced reliability from phase II study	Inclusion of comparator arm in this setting and also randomization in phase II studies [91].
A large number of patients are required for randomization in an early setting	Bayesian models with flexible and adaptive trial designs offer to test more compounds at the same time (comparing them to a shared comparator arm) with a reduced number of patients [92].
A long time from the trial start to the final result	(1) Bayesian adaptive randomized (AR) studies [93,94,95] (2) Use of different endpoints such as a composed PFS-OS endpoint or ORR through assessment of learning algorithms [96].
A large number of novel compounds in pre-clinical phases	Phase 0 trials [97].
Molecular heterogeneity of the disease	Umbrella trial in which treatment arm allocation is driven by the molecular composition of the disease [98].
